# Molecular Characterization and Antibiogram Profiling of Bacteria Isolated From Sewage and Surface Water in Bangladesh

**DOI:** 10.1155/sci5/1848058

**Published:** 2025-09-03

**Authors:** Md. Arif-Uz-Zaman Polash, Md. Shamsul Islam, Nusrat Zahan, Subir Sarker, Md. Hakimul Haque

**Affiliations:** ^1^Department of Veterinary and Animal Sciences, University of Rajshahi, Rajshahi 6205, Bangladesh; ^2^Biomedical Sciences & Molecular Biology, College of Medicine and Dentistry, James Cook University, Townsville, Queensland 4811, Australia; ^3^Australian Institute of Tropical Health and Medicine, James Cook University, Townsville, Queensland 4811, Australia; ^4^Australian Institute for Bioengineering and Nanotechnology (AIBN), The University of Queensland, Brisbane, Queensland 4072, Australia

**Keywords:** antibiogram, bacteria isolation, Bangladesh, molecular detection, sewage, surface water

## Abstract

The global rise of antibiotic-resistant bacteria presents a major threat to public health, complicating the treatment of bacterial infections. This study aimed to identify bacterial pathogens in surface water and sewage samples from the University of Rajshahi, Bangladesh, and evaluate their antibiotic susceptibility. A total of 60 water samples were collected from four distinct locations and analyzed using a combination of culture-based techniques, conventional PCR, and advanced molecular techniques (Sanger sequencing). Eight prevalent bacterial species were identified: *Klebsiella pneumoniae* (21.6%), *Escherichia fergusonii* (15%), *Enterobacter bugandensis* (13.3%), *Bacillus paramycoides* (8.3%), *Comamonas jiangduensis* (8.3%), *Bacillus albus* (6.6%), *Klebsiella quasivariicola* (5%), and *Lysinibacillus xylanilyticus* (5%). The 16S rRNA gene sequencing confirmed the identity of the bacterial isolates, and the phylogenetic tree analysis revealed distinct genetic divergence of the Bangladeshi isolates compared to global reference strains. Antibiotic susceptibility against 10 commonly used antibiotics was performed using the Kirby–Bauer disk diffusion method, revealing a varying degree of resistance patterns. All isolated bacteria exhibited susceptibility to imipenem, levofloxacin, amikacin, and azithromycin, while significant resistance was noted against cefradine, amoxicillin/clavulanic acid, cefuroxime, and ceftriaxone. Notably, 44% of the bacterial isolates were identified as multi–drug-resistant (MDR), with *K. pneumoniae* (69.23%), *E. bugandensis* (62.5%), and *E. fergusonii* (55.55%) exhibiting the highest resistance. In contrast, *K. quasivariicola* and *C. jiangduensis* exhibited no MDR traits. The multiple antibiotic resistance (MAR) index ranged from 0.30 to 0.60 among the isolates. These findings highlight the significant contamination of water sources with antibiotic-resistant bacteria, underscoring the urgent need for effective management practices to mitigate public health risks.

## 1. Introduction

Water is one of the most critical resources on Earth, essential for all forms of life. Unfortunately, water quality is severely compromised in many parts of the world due to contamination by chemical and biological pollutants [1]. This is particularly prevalent in water bodies contaminated with human waste, including feces and urine, leading to the proliferation of harmful bacteria [2]. These waterborne pathogens pose significant health risks to individuals who come into contact with contaminated water through activities such as swimming, bathing, or consuming food prepared with polluted water [3]. The global burden of waterborne illnesses is especially pronounced in developing countries, where access to safe water is limited, resulting in a high incidence of disease and mortality among children and adults [4].

Water serves as a critical environment for microbial communities, allowing bacterial interactions across diverse sources and enabling the transfer of genes, including those responsible for antibiotic resistance [5, 6]. The components of water, particularly in areas where it is untreated or improperly managed, contribute to the selection of antibiotic-resistant bacteria, posing a significant threat to public health [7]. Contamination of surface and sewer waters with pathogenic bacteria is a persistent issue [8], adversely affecting water quality and contributing to the spread of waterborne diseases [9–11]. For example, a recent study in China highlighted the prevalence of Proteobacteria and Bacteroidetes, with potentially hazardous bacteria found across all urban surface water samples examined [12]. Diarrhea, often caused by ingesting microorganisms in water polluted with fecal matter, is a common consequence of such contamination, leading to millions of cases annually, particularly in regions with inadequate water and sanitation infrastructure [13, 14].

Water is a significant reservoir for dangerous bacteria, including those carrying antibiotic-resistant genes [2]. These bacteria can be introduced into aquatic ecosystems through discharges from municipal systems, pharmaceutical facilities, and agricultural runoff [15–17]. These bacteria can spread antibiotic resistance, complicating the treatment of infectious diseases and leading to the emergence of multidrug-resistant (MDR) organisms [18]. This trend presents a grave threat to public health, including prolonged hospital stays, extended treatment courses, and, in some cases, treatment failure [19, 20]. The presence of MDR bacteria in various water sources, such as drinking water, surface water, groundwater, and wastewater, underscores the role of aquatic environments as reservoirs for these pathogens [15–17].

Given the rapid spread of antibiotic-resistant bacteria, there is an urgent need to understand the epidemiology of drug-resistant microbes in water sources. The World Health Organization (WHO) has underscored the importance of addressing antimicrobial resistance, warning that it could lead to millions of deaths and significant economic losses by 2050 if left unaddressed [21]. Several studies have assessed water quality in Bangladesh by quantifying pathogenic bacteria, particularly fecal coliforms [22, 23]. Research from various countries, such as India, Pakistan, Iraq, Türkiye, and Nigeria, has documented the presence of MDR bacteria in different water sources. These studies showed that aquatic environments can serve as reservoirs for bacteria that are resistant to antibiotics, like *Escherichia fergusonii* [24], *Comamonas* spp. and *Bacillus* spp. [25], *Bacillus paramycoides* [26], *Klebsiella pneumoniae* [27], *Klebsiella quasivariicola* [28], *Enterobacter bugandensis* [29], *Lysinibacillus* spp. [30], and *Comamonas jiangduensis* [31]. Therefore, this study aims to investigate the molecular detection and antibiogram profiling of bacteria in sewage and surface water. By examining bacterial contamination and antibiotic resistance in these water sources, this research seeks to inform public health strategies to mitigate the risks associated with waterborne diseases, particularly in developing countries where the disease burden is most severe [32].

## 2. Materials and Methods

### 2.1. Sample Collection and Processing

The experimental procedures and protocols used for this study were approved by the Institutional Animal, Medical Ethics, Biosafety, and Biosecurity Committee of the Institute of Biological Science at the University of Rajshahi (Memo no. 56/321/IAMEBBC/IBSc). A total of 60 water samples were collected from four different locations at the University of Rajshahi, Bangladesh, following standard procedures using clean, sterile plastic containers between September and October 2023. These locations included pond water, swimming pool water as surface water, and sewage water from the boys' and girls' residential dormitories. Fifteen samples were taken from each site, encompassing both surface water and sewage water. The samples were then transported to the Department of Veterinary and Animal Sciences at Rajshahi University, adhering to sterile and cold chain conditions to ensure microbiological analysis within less than an hour. All sample collection and processing procedures were conducted under the relevant guidelines and regulations.

### 2.2. Isolation and Characterization of Bacteria

Bacterial isolation and identification were conducted by culturing surface water and sewage samples on MacConkey and HiCrome UTI Agar (HiMedia, India) plates following initial cultivation in nutrient broths, which were incubated aerobically at 37°C overnight to observe specific colony characteristics. Among the isolates, *Comamonas jiangduensis*, *Klebsiella pneumoniae*, *Klebsiella quasivariicola*, *Escherichia fergusonii*, and *Enterobacter bugandensis* produced pale to pink colonies on MacConkey agar, while *Bacillus albus*, *Bacillus paramycoides*, and *Lysinibacillus xylanilyticus* showed no growth. On HiCrome UTI Agar, the isolated bacteria exhibited a range of colony morphologies: *Comamonas jiangduensis* and *Enterobacter bugandensis* formed greenish colonies, *Klebsiella pneumoniae* produced blue mucoid colonies, *Escherichia fergusonii* appeared as pinkish purple colonies, *Bacillus albus* and *Bacillus paramycoides* exhibited creamy white colonies, and *Lysinibacillus xylanilyticus* along with *Klebsiella quasivariicola* displayed purple colonies. Further characterization of the colonies' morphology and biochemical properties was carried out using Gram staining, sugar fermentation tests, catalase test, methyl red tests, Voges–Proskauer tests, TSI agar reaction tests, and Indole tests, in accordance with methods described by Bergey [33].

### 2.3. Bacterial Genomic DNA Extraction

The genomic DNA was extracted from pure cultures by the boiling method, as described by Mahmud et al. [34]. Briefly, 200 μL of deionized ultrapure water was placed in a sterile Eppendorf tube, and a pure colony of the isolated bacteria was added from the overnight culture on nutrient agar at 37°C. The mixture was gently vortexed, followed by boiling, and cooling for 10 min each. After centrifugation at 1000 rpm for 10 min, 100 μL of the supernatant containing the genomic DNA was collected. The pureness and quantity of the extracted DNA were assessed using a NanoDrop spectrophotometer (BioLab, Ipswich, MA, USA), and the DNA was then kept at −20°C for further use.

### 2.4. Polymerase Chain Reaction (PCR) and Sequencing

To confirm the identity of the isolates, PCRs were performed targeting the near-complete region of 16S rRNA gene for each isolate using a series of primers (5′-AGAGTTTGATCCTGGCTCAG-3′) and 1429R (5′-GGTTACCTTGTTACGACTT-3′) as described by McCabe et al. [35]. The PCR was carried out in a total volume of 25 μL, which included 12.5 μL of PCR Master Mix (2x) (Thermo Scientific, USA), 1 μL of 10 pmol of each primer, 2 μL of template (DNA extract at 50 ng/μL), and 9.5 μL of nuclease-free water. Following an initial denaturation at 95°C for 5 min, the samples underwent 35 cycles of denaturation at 94°C for 30 s, annealing at 57°C for 30 s, and extension at 72°C for 1 min, with a final extension at 72°C for 10 min. The amplification products were analyzed by electrophoresis on a 1.5% agarose gel, and the amplicons were visualized using ethidium bromide under an ultraviolet transilluminator (Biometra, Germany). A 1-kb DNA ladder (Promega, USA) was used as a molecular weight marker. Sanger sequencing was conducted on a total of eight samples of each conventionally detected isolate for further confirmation of bacterial species. The amplified and specific PCR bands were excised and purified using the procedures outlined in the NucleoSpin Gel and PCR Clean-up kit (Macherey-Nagel, Bethlehem, PA, USA). Purified PCR products (10–40 ng of DNA) were mixed with 1 μL of 3.2 pmol primers in 10 μL of H_2_O using Big Dye Terminator (BDT) chemistry Version 3.1 (Applied Biosystems). Sanger sequencing was performed and analyzed using an ABI PRISM 3730xl Capillary sequencer (Applied Biosystems, USA) under standardized cycling PCR conditions. Single-end sequences were aligned using MEGA11 software and blasted against the EMBL-EBI database to identify the phylogeny of the isolates.

### 2.5. Accession Numbers for Nucleotide Sequences

The 16S rRNA gene nucleotide sequences of *Comamonas jiangduensis*, *Escherichia fergusonii*, *Enterobacter bugandensis*, *Klebsiella pneumoniae*, *Klebsiella quasivariicola*, *Bacillus albus*, *Bacillus paramycoides*, and *Lysinibacillus xylanilyticus* from this investigation have been submitted to the National Center for Biotechnology Information (NCBI, Bethesda, MD, USA) under the following accession numbers: PP813580.1, PP813627.1, PP813633.1, PP813620.1, PP813623.1, PP817696.1, PP813619.1, and PP813624.1, respectively.

### 2.6. Phylogenetic Tree Construction

The sequences of the 16S rRNA gene (27F–1492R) were blasted against NCBI GenBank database and showed 100% identity with the assumed species based on morphology. The neighbor-joining method [36] was used to determine the evolutionary relationships among the bacterial isolates under this study. The maximum composite likelihood method [37] was employed to calculate the average number of base changes per site, providing insights into the evolutionary divergence between organisms. All steps in the evolutionary analysis were performed using MEGA11 software.

### 2.7. Antimicrobial Susceptibility Test (AST)

All the bacteria isolated from surface water and sewage samples were subjected to ASTs using the Kirby–Bauer disk diffusion method [38]. Ten commonly used antibiotics from six classes were tested, including fluoroquinolones (levofloxacin 5 μg, ciprofloxacin 5 μg), aminoglycosides (amikacin 30 μg, gentamicin 10 μg), cephalosporins (ceftriaxone 30 μg, cefuroxime 30 μg, cefradine 30 μg), macrolides (azithromycin 15 μg), penicillin (amoxicillin + clavulanic acid 30 μg), and carbapenems (imipenem 10 μg). ASTs were conducted on Mueller–Hinton agar plates (HiMedia, India) using freshly cultured bacteria at a density of 0.5 McFarland units. The outcomes were classified as sensitive or resistant (Table S1) following the references of the Clinical and Laboratory Standards Institute [39]. MDR isolates were categorized according to the method proposed by Sweeney et al. [40]. Additionally, the multiple antibiotic resistance (MAR) index was calculated using the formula MAR = *a*/*b*, where “*a*” represents the number of drugs to which a specific isolate showed resistance and “*b*” denotes the total number of antibiotics examined [41].

### 2.8. Statistical Analysis

Microsoft Excel 2010 was used for data entry, and IBM SPSS Version 24 (Armonk, NY, USA) was employed for the subsequent analysis. Occurrence rates were determined using descriptive statistics, and statistical significance was assessed with a *p*-value threshold of less than 0.05.

## 3. Results

### 3.1. Prevalence of Bacteria in Surface Water and Sewage Samples

A bacteriological analysis was conducted on 60 water samples collected from four distinct sites at the University of Rajshahi. Out of these 60 samples, 50 (83.33%) tested positive for eight different bacterial species, with prevalence rates ranging from 5% (*Klebsiella quasivariicola*, *Lysinibacillus xylanilyticus*) to 21.6% (*Klebsiella pneumoniae*), as detailed in Table 1. These bacteria were further confirmed through isolation on selective HiCrome UTI Agar, Gram staining, cultural characteristics, and biochemical assays. 60 water samples (the highest number of samples) tested positive (*n* = 13, 21.6%) for *Klebsiella pneumoniae*, followed by *Escherichia fergusonii* (*n* = 9, 15%), and *Enterobacter bugandensis* (*n* = 8, 13.3%) (Table 1). The occurrence of *Klebsiella pneumoniae* was notably higher in swimming pool water and sewage from the girls' residential dormitory, with each showing 26.6% instances. This was followed by pond water with 20% instances. The lowest occurrence was in boys' residential dormitory sewage water with 13.3% instances (Table 1). *Escherichia fergusonii* was most commonly found in the sewage water of both boys' and girls' residential dormitories, with 20% instances each, followed by pond water with 2 (13.3%) instances, and swimming pool water with 1 (6.6%) instance. *Enterobacter bugandensis* was most prevalent in boys' residential dormitory sewage water with 3 (20%) instances, followed by swimming pool water and girls' residential dormitory sewage water, each with 2 (13.3%) instances, and pond water with 1 (6.6%) instance. *Comamonas jiangduensis* was most frequently found in pond water with 2 (13.3%) instances, followed by swimming pool water, boys' residential dormitory sewage water, and girls' residential dormitory sewage water, each with 1 (6.6%) instance. The highest occurrence of *Bacillus paramycoides* was in girls' residential sewage water with 2 (13.3%) instances, followed by pond water, swimming pool water, and boys' residential dormitory sewage water, each with 1 (6.6%) instance. *Bacillus albus* was most prevalent in boys' residential dormitory sewage water with 2 (13.3%) instances, followed by swimming pool water and girls' residential dormitory sewage water, each with 1 (6.6%) instance, while it was absent in pond water. *Klebsiella quasivariicola* and *Lysinibacillus xylanilyticus* were equally distributed across pond water, swimming pool water, and boys' residential dormitory sewage water, with 1 (6.6%) instance each, but were not detected in girls' residential dormitory sewage water. These findings indicate that the differences in the presence of these bacteria across surface and sewage water samples from the University of Rajshahi community are not statistically significant (*p*=0.996) (Table 1).

### 3.2. Confirmation of Isolated Bacteria by 16S rRNA and Sequencing

The amplified 16S rRNA gene resulted in 1400 base pair fragments (Figure S1), which were further confirmed by Sanger sequencing. Results revealed fragment lengths of 947 bp for *Klebsiella pneumoniae*, 1052 bp for *Klebsiella quasivariicola*, 1273 bp for *Bacillus albus*, 774 bp for *Bacillus paramycoides*, 730 bp for *Lysinibacillus xylanilyticus*, 880 bp for *Enterobacter bugandensis*, 961 bp for *Escherichia fergusonii*, and 757 bp for *Comamonas jiangduensis*. Analysis of the sequence data showed the highest sequence similarities of 94.81%, 96.98%, 99.66%, 97.80%, 99.04%, 98.75%, 97.81%, and 81.61%, respectively, with the reference strains: *Klebsiella pneumoniae* strain ATCC 13883, *Klebsiella quasivariicola* strain KPN 1705, *Bacillus albus* strain MCCC 1A02146, *Bacillus paramycoides* strain MCCC 1A04098, *Lysinibacillus xylanilyticus* strain XDB 9, *Enterobacter bugandensis* strain 247 BMC, *Escherichia fergusonii* strain ATCC 35469, and *Comamonas jiangduensis* strain YW 1.

### 3.3. Phylogenetic Analysis of the Isolated Bacteria

**Figure 1:**
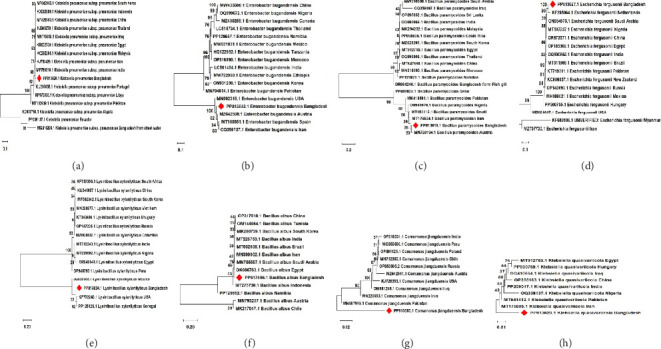
Phylogenetic tree constructed using the 16S ribosomal RNA gene sequences from various bacterial species, including (a) *Klebsiella pneumoniae*; (b) *Enterobacter bugandensis*; (c) *Bacillus paramycoides*; (d) *Escherichia fergusonii*; (e) *Lysinibacillus xylanilyticus*; (f) *Bacillus albus*; (g) *Comamonas jiangduensis*; and (h) *Klebsiella quasivariicola*, sourced from different countries. The red bullet point indicates that the isolates are from Bangladesh. Varying length scale bars were used to construct these phylogenetic trees, which ranged from 0.01 to 0.20 substitutions per site.

To elucidate the evolutionary relationships of the newly sequenced 16S rRNA genes of bacteria identified in water samples from the RU community in Bangladesh, phylogenetic trees were constructed for each newly identified bacterial species to compare them with similar species isolated from different countries.  Figure 1 illustrates the phylogenetic trees, highlighting the genetic divergence between the Bangladeshi isolates and those from other regions. Notably, the phylogenetic analysis indicates that the bacterial strains isolated in Bangladesh are genetically distinct from other global isolates of the same species.

### 3.4. Antibiogram Profile of Isolated Bacteria

**Figure 2:**
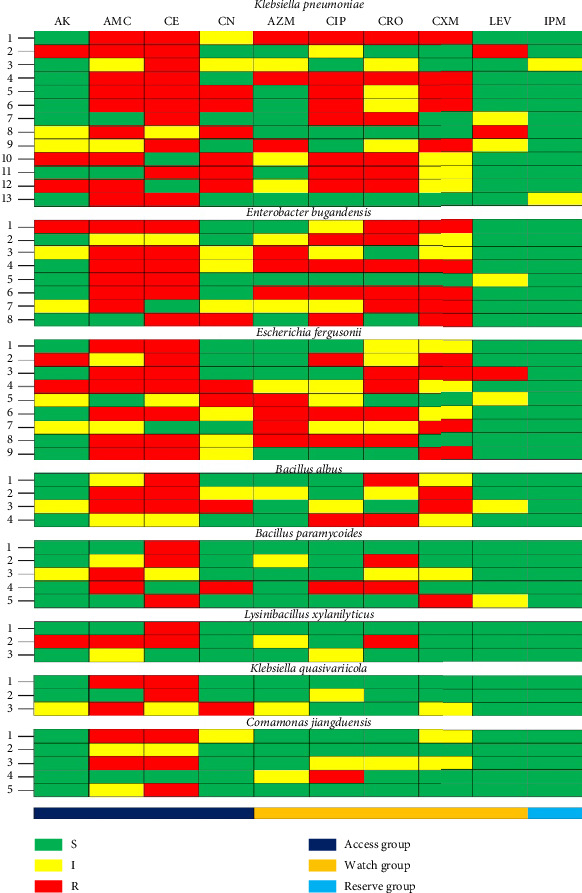
Heat map exhibiting the antibiogram profiles of *Klebsiella pneumoniae*, *Enterobacter bugandensis*, *Escherichia fergusonii*, *Bacillus albus*, *Bacillus paramycoides*, *Lysinibacillus xylanilyticus*, *Klebsiella quasivariicola*, and *Comamonas jiangduensis* isolated from water samples from four distinct sites at the University of Rajshahi, Bangladesh: AK = amikacin, AMC = amoxicillin + clavulanic acid, CE = cefradine, CN = gentamicin, AZM = azithromycin, CIP = ciprofloxacin, CRO = ceftriaxone, CXM = cefuroxime, LEV = levofloxacin, IPM = imipenem, S = sensitive, I = intermediate, R = resistant.

The antibiotic susceptibility test was conducted using the disk diffusion method on a panel of 10 commonly prescribed antibiotics from six different classes. The results revealed that all tested bacterial isolates exhibited resistance to multiple antibiotics (Figure 2 and Supporting Tables S2–S9). *Klebsiella pneumoniae* strains showed significant resistance to cefradine (76.92%), amoxicillin/clavulanic acid (69.23%), and ciprofloxacin (61.53%), while they were highly sensitive to imipenem (100%), levofloxacin (84.61%), amikacin (76.93%), azithromycin (76.93%), and cefuroxime (61.53%). For *Klebsiella quasivariicola*, resistance was noted against amoxicillin/clavulanic acid (66.66%), cefradine (66.66%), and gentamicin (33.33%). In contrast, this species exhibited 100% sensitivity to ceftriaxone, levofloxacin, and imipenem. *Bacillus albus* strains showed resistance to cefradine (75%), amoxicillin/clavulanic acid (50%), ceftriaxone (50%), and cefuroxime (50%). However, they were fully sensitive to imipenem, levofloxacin, amikacin, and azithromycin, but only 75% sensitive to ciprofloxacin and gentamicin. *Bacillus paramycoides* strains were resistant to cefradine (60%), but 100% sensitive to imipenem, levofloxacin, amikacin, and azithromycin. For *Lysinibacillus xylanilyticus*, resistance to cefradine (66.66%) was observed, while imipenem, levofloxacin, azithromycin, cefuroxime, ciprofloxacin, and gentamicin were 100% effective. *Enterobacter bugandensis* strains exhibited resistance to amoxicillin/clavulanic acid and cefradine (75% each), as well as ceftriaxone and cefuroxime (62.5% each). Imipenem and levofloxacin were 100% effective, followed by amikacin and gentamicin (87.5% each) and azithromycin (62.5%). *Escherichia fergusonii* strains demonstrated resistance to cefradine (77.77%) and amoxicillin/clavulanic acid (66.66%). However, imipenem showed 100% effectiveness, with levofloxacin (88.88%), amikacin (77.77%), gentamicin (77.77%), and ciprofloxacin (66.66%) also exhibiting notable effectiveness. *Comamonas jiangduensis* strains were resistant to cefradine (60%), amoxicillin/clavulanic acid (40%), and ciprofloxacin (20%). They were fully sensitive to imipenem, levofloxacin, amikacin, azithromycin, ceftriaxone, cefuroxime, and gentamicin. Ciprofloxacin showed 80% effectiveness, while amoxicillin/clavulanic acid was 60% effective.

### 3.5. Occurrence of MDR Patterns and MAR Index of Isolated Bacteria

Out of the 13 *Klebsiella pneumoniae* isolates, 9 (69.23%) exhibited an MDR phenotype. Six distinct resistance patterns were observed among these isolates. The most common pattern, exhibited by 22.22% (2/9) of the MDR isolates, was pattern no. 1 (AMC, CE, AZM, CIP, CRO, CXM), followed by pattern no. 2 (AMC, CE, CN, CIP, CXM), and pattern no. 3 (AK, AMC, CN, CIP, CRO). The MAR index for each *Klebsiella pneumoniae* isolate ranged from 0.30 to 0.60. In the case of *Enterobacter bugandensis*, 5 out of 8 isolates (62.5%) displayed an MDR phenotype, with four distinct resistance patterns identified. The most prevalent pattern, observed in 40% (2/5) of the MDR isolates, was pattern no. 1 (AMC, CE, AZM, CIP, CRO, CXM). The MAR index for *Enterobacter bugandensis* isolates ranged from 0.30 to 0.60. Among the 9 *Escherichia fergusonii* isolates, 5 (55.55%) exhibited an MDR phenotype. Four distinct resistance patterns were documented, with the highest proportion 40% (2/5) displaying pattern no. 1 (AMC, CE, AZM, CIP, CRO). The MAR index for *Escherichia fergusonii* isolates ranged from 0.40 to 0.50. For *Bacillus albus*, 1 out of 4 isolates (25%) exhibited an MDR phenotype. This isolate displayed resistance to pattern no. 1 (AMC, CE, CN, CXM), with a MAR index of 0.50. Similarly, 1 out of 5 *Bacillus paramycoides* isolates (20%) demonstrated an MDR phenotype, with resistance to pattern no. 1 (AMC, CN, CIP, CRO) and a MAR index of 0.40. Additionally, 1 out of 3 *Lysinibacillus xylanilyticus* isolates (33.33%) exhibited an MDR phenotype, showing resistance to pattern no. 1 (AK, AMC, CE, CRO), with a MAR index of 0.40. Interestingly, none of the *Klebsiella quasivariicola* and *Comamonas jiangduensis* isolates demonstrated an MDR phenotype during antibiotic susceptibility testing using the disk diffusion method (Table 2).

## 4. Discussion

The identification of bacterial pathogens and their corresponding antibiogram profiles is crucial for managing infections in both rural and urban communities, as well as in healthcare settings. This study revealed significant bacterial contamination in water samples from ponds, swimming pools, and sewage at the University of Rajshahi, with eight distinct species identified (Table 1). The presence of these bacteria in sewage is expected due to human waste inputs, but their detection in pond and swimming pool water raises serious public health concerns. This contamination likely stems from untreated sewage entering water bodies and inadequate water purification regulations in Bangladesh, particularly for swimming pools, which were not sufficiently monitored for water quality. Our study found that 83.3% of tested water samples were positive for bacterial presence. Interestingly, a study by Ibrahim and Hameed in Baghdad, Iraq, reported that *Klebsiella pneumoniae* was the most predominant pathogen in environmental samples, with an incidence rate of 32.8% [27]. In contrast, Singh et al. found a higher prevalence of *Escherichia fergusonii* in spring water in Sikkim, India, with a prevalence rate of 29.41% [24]. Another study by Koskeroglu et al. revealed a lower prevalence of *Enterobacter bugandensis* (6.6%) among 70 isolates identified from 300 water samples collected from various water bodies, including surface water, pools, and drinking water [29]. Additionally, Fiaz et al. reported the prevalence rates of *Comamonas* spp. (2.7%), *Escherichia* spp. (6.4%), and *Bacillus* spp. (1%) isolated from wastewater in Pakistan [25]. Obayiuwana et al. found the prevalence rates of *Enterobacter* sp. (9.45%), *Bacillus* sp. (7%), and *Lysinibacillus* sp. (1.2%) in wastewater from pharmaceutical facilities in Lagos and Ogun States, Nigeria [30]. The variation in the incidence rates of *Klebsiella pneumoniae*, *Klebsiella quasivariicola*, *Bacillus albus*, *Bacillus paramycoides*, *Lysinibacillus xylanilyticus*, *Enterobacter bugandensis*, *Escherichia fergusonii*, and *Comamonas jiangduensis* could be attributed to several factors. These include differences in sample collection methods, geographical location, the availability of species identification techniques in laboratories, the timing of sample collection, and the lack of advanced technology in many developing countries like Bangladesh. However, these findings align with similar studies conducted in India, Pakistan, Iraq, Türkiye, and Nigeria [24–30]. These studies suggest variations in the prevalence rates of bacteria in water may be partly due to variances in sample size, adherence to water bacteria isolation guidelines, and the presence or absence of wastewater treatment procedures.

In this study, PCR amplification and phylogenetic analysis of the 16S rRNA gene checked that the isolates belong to species such as *Klebsiella pneumoniae*, *Klebsiella quasivariicola*, *Bacillus albus*, *Bacillus paramycoides*, *Lysinibacillus xylanilyticus*, *Enterobacter bugandensis*, *Escherichia fergusonii*, and *Comamonas jiangduensis* [42]. In Bangladesh, bacterial species identification is often overlooked before conducting ASTs. This study highlights the significance of precise bacterial identification and sets a precedent for detecting commensal bacteria from environmental samples, potentially improving the accuracy of diagnostic and treatment approaches in the region. For instance, studies in India [24] and Nigeria [30] have similarly emphasized the importance of molecular techniques for identifying waterborne pathogens, highlighting their role in informing public health interventions.

Antibiotic resistance poses an escalating threat to public health worldwide, necessitating immediate and comprehensive responses [43]. This study identified significant MDR phenotypes among the isolated bacteria, with high resistance rates observed for *Klebsiella pneumoniae*, *Enterobacter bugandensis*, and *Escherichia fergusonii* (Table 2).

These findings underscore the growing resistance among various bacterial isolates. A notable concern is the high prevalence of MDR strains found in this study, with 44% of bacterial isolates demonstrating MDR, comparable to rates reported in South Romania (37.94%) and Pakistan (> 30%) [25, 44]. However, this figure is lower than the 87.8% reported in the Czech Republic and 85.5% in Nigeria [30, 45]. These variations in MDR incidence may be influenced by factors such as the time period of the research, differences in sample size, and geographical factors. In contrast, previous research has reported higher MDR rates in these species, such as 75.93% MDR in *E. fergusonii* isolated from poultry in China [46–48]. The emergence of MDR strains in pathogens like *Enterobacter bugandensis* (62.5%) and *Lysinibacillus xylanilyticus* (33.33%) signals an urgent need for enhanced antimicrobial stewardship. The findings also revealed MAR indices ranging from 0.30 to 0.60 among the bacterial isolates, except for *K. quasivariicola* and *Comamonas jiangduensis*. This aligns with findings from Singh et al. (2020), who reported elevated MDR rates in *Escherichia fergusonii* from Indian spring water [24], and Obayiuwana et al., who noted similar trends in Nigerian pharmaceutical wastewater [30].

The emergence of MDR strains, particularly in pathogens like *Enterobacter bugandensis* and *Lysinibacillus xylanilyticus*, underscores the need for enhanced antimicrobial stewardship and stricter regulations on antibiotic use. Potential mechanisms, such as beta-lactamase production in *Klebsiella pneumoniae* and *Enterobacter bugandensis* or horizontal gene transfer in aquatic environments, may drive these resistance patterns, warranting further genomic studies to elucidate resistance mechanisms and transmission pathways. This study has several limitations. The use of culture-based methods may underestimate bacterial diversity, as nonculturable or fastidious species may not be detected. Additionally, sampling was conducted over a limited period (September–October 2023), which may not capture seasonal variations in bacterial prevalence or resistance. The focus on four specific sites at the University of Rajshahi may also limit the generalizability of findings to broader aquatic environments. Future research could employ metagenomic sequencing and extended sampling periods to address these constraints.

## 5. Conclusion

This study highlights the alarming presence of multiple antibiotic-resistant bacteria in the water of the University of Rajshahi, raising potential health risks for the community that relies on this water for daily activities. Notably, all the isolated bacteria showed high susceptibility to imipenem, levofloxacin, amikacin, and azithromycin, but showed significant resistance to cefradine, amoxicillin/clavulanic acid, cefuroxime, and ceftriaxone. The high rate of antimicrobial resistance among environmental bacteria could result from indiscriminate use of antibiotics in human, animal, and agricultural sectors. The study emphasizes the urgent threat posed by antibiotic-resistant bacteria in the region, which calls for effective measures, including stricter regulations on antibiotic use, comprehensive surveillance of waterborne pathogens, and broader public education. Additionally, further research is essential to better understand the health risks associated with exposure to contaminated water and to develop strategies to mitigate this emerging public health problem.

## Figures and Tables

**Table 1:** Number of positive bacteria in surface and sewage water samples from different sources at the University of Rajshahi.

Isolates	Pond (*n* = 15)	Swimming pool (*n* = 15)	Boys' residential dormitory (*n* = 15)	Girls' residential dormitory (*n* = 15)	Total	Chi-square	*p* value
*Klebsiella pneumoniae*	3 (20%)	4 (26.6%)	2 (13.3%)	4 (26.6%)	13 (21.6%)	7.851	0.996
*Klebsiella quasivariicola*	1 (6.6%)	1 (6.6%)	1 (6.6%)	0 (0%)	3 (5%)		
*Bacillus albus*	0 (0%)	1 (6.6%)	2 (13.3%)	1 (6.6%)	4 (6.6%)		
*Bacillus paramycoides*	1 (6.6%)	1 (6.6%)	1 (6.6%)	2 (13.3%)	5 (8.3%)		
*Lysinibacillus xylanilyticus*	1 (6.6%)	1 (6.6%)	1 (6.6%)	0 (0%)	3 (5%)		
*Enterobacter bugandensis*	1 (6.6%)	2 (13.3%)	3 (20%)	2 (13.3%)	8 (13.3%)		
*Escherichia fergusonii*	2 (13.3%)	1 (6.6%)	3 (20%)	3 (20%)	9 (15%)		
*Comamonas jiangduensis*	2 (13.3%)	1 (6.6%)	1 (6.6%)	1 (6.6%)	5 (8.3%)		

**Table 2:** Occurrence of multidrug resistance and multiple antibiotic resistance indexes of *Klebsiella pneumoniae*, *Enterobacter bugandensis*, *Escherichia fergusonii*, *Bacillus albus*, *Bacillus paramycoides*, and *Lysinibacillus xylanilyticus* in different water samples.

Name of bacteria	Pattern no.	Antibiotic resistance patterns	No. of antibiotic (classes)	No. of MDR isolates (%)	Overall MDR isolates (%)	MAR index
*Klebsiella pneumoniae* (*n* = 13)	1	AMC, CE, AZM, CIP, CRO, CXM	6 (4)	2 (22.22%)	9 (69.23%)	0.60
	2	AMC, CE, CN, CIP, CXM	5 (4)	2 (22.22%)		0.50
	3	AK, AMC, CN, CIP, CRO	5 (4)	2 (22.22%)		0.50
	4	AK, AMC, CE, LEV	4 (4)	1 (11.11%)		0.40
	5	CE, CN, CIP, CRO	4 (3)	1 (11.11%)		0.40
	6	AMC, CN, LEV	3 (3)	1 (11.11%)		0.30

*Enterobacter bugandensis* (*n* = 8)	1	AMC, CE, AZM, CIP, CRO, CXM	6 (4)	2 (40%)	5 (62.5%)	0.60
	2	AK, AMC, CE, CRO, CXM	5 (3)	1 (20%)		0.50
	3	CE, CN, CIP, CXM	4 (3)	1 (20%)		0.40
	4	AMC, CE, AZM	3 (3)	1 (20%)		0.30

*Escherichia fergusonii* (*n* = 9)	1	AMC, CE, AZM, CIP, CRO	5 (4)	2 (40%)	5 (55.55%)	0.50
	2	AMC, CE, CRO, CXM, LEV	5 (3)	1 (20%)		0.50
	3	AK, AMC, CE, CN, CRO	5 (3)	1 (20%)		0.50
	4	AK, CE, CIP, CXM	4 (3)	1 (20%)		0.40

*Bacillus albus* (*n* = 4)	1	AMC, CE, CN, CXM	4 (3)	1 (25%)	1 (25%)	0.50

*Bacillus paramycoides* (*n* = 5)	1	AMC, CN, CIP, CRO	4 (4)	1 (20%)	1 (20%)	0.40

*Lysinibacillus xylanilyticus* (*n* = 3)	1	AK, AMC, CE, CRO	4 (3)	1 (33.33%)	1 (33.33%)	0.40

*Note:* AK = amikacin, AMC = amoxicillin/clavulanic acid, AZM = azithromycin, CE = cefradine, CRO = ceftriaxone, CXM = cefuroxime, CIP = ciprofloxacin, CN = gentamicin, IPM = imipenem, LEV = levofloxacin.

Abbreviations: MAR = multiple antibiotic resistance, MDR = multi–drug-resistant.

## Data Availability

All authors of the study had full access to the data.

## Supporting Information

Figure S1. Amplification of the 16S rRNA gene in a 1.5% agarose gel. The 16S rRNA gene is present in all samples (Lanes 2–9). A 1-kb DNA ladder was used as a molecular weight marker for comparison.

Table S1. Antimicrobial with zone of standard in diameter (according to the CLSI, 2016).

Table S2. Results of antimicrobial sensitivity and resistant pattern of *Klebsiella pneumoniae*.

Table S3. Results of antimicrobial sensitivity and resistant pattern of *Klebsiella quasivariicola*

Table S4. Results of antimicrobial sensitivity and resistant pattern of *Bacillus albus*.

Table S5. Results of antimicrobial sensitivity and resistant pattern of *Bacillus paramycoides*

Table S6. Results of antimicrobial sensitivity and resistant pattern of *Lysinibacillus xylanilyticus*.

Table S7. Results of antimicrobial sensitivity and resistant pattern of *Enterobacter bugandensis*.

Table S8. Results of antimicrobial sensitivity and resistant pattern of *Escherichia fergusonii*.

Table S9. Results of antimicrobial sensitivity and resistant pattern of *Comamonas jiangduensis*

## References

[1] Kumar L., Kumari R., Kumar A., Tunio I. A., Sassanelli C. (2023). Water Quality Assessment and Monitoring in Pakistan: A Comprehensive Review. *Sustainability*.

[2] Samrot A. V., Wilson S., Sanjay Preeth R. S. (2023). Sources of Antibiotic Contamination in Wastewater and Approaches to Their removal—An Overview. *Sustainability*.

[3] Mudau M., Ngobeni-Nyambi R., Momba M. N. B. (2023). The Fascinating Cross-Paths of Pathogenic Bacteria, Human and Animal Faecal Sources in Water-Stressed Communities of Vhembe District, South Africa. *Pathogens*.

[4] Magana-Arachchi D. N., Wanigatunge R. P. (2020). *Ubiquitous Waterborne Pathogens*.

[5] Baquero F., Martínez J. L., Canton R. (2008). Antibiotics and Antibiotic Resistance in Water Environments. *Current Opinion in Biotechnology*.

[6] Manaia C. M., Macedo G., Fatta-Kassinos D., Nunes O. C. (2016). Antibiotic Resistance in Urban Aquatic Environments: Can It Be Controlled?. *Applied Microbiology and Biotechnology*.

[7] Adekanmbi A. O., Osuzoka I. A., Aremu O., Olaposi A. (2020). Antibiogram and Non-Detection of mecA Gene in *Staphylococcus* spp. Isolated From a Sewage-Imparted Stream Within a University Community. *MicroMedicine*.

[8] Bej S., Swain S., Bishoyi A. K., Mandhata C. P., Sahoo C. R., Padhy R. N. (2023). Wastewater-Associated Infections: A Public Health Concern. *Water, Air, & Soil Pollution*.

[9] Titilawo Y., Sibanda T., Obi L., Okoh A. (2015). Multiple Antibiotic Resistance Indexing of *Escherichia coli* to Identify High-Risk Sources of Faecal Contamination of Water. *Environmental Science and Pollution Research*.

[10] Breidenstein E. B., Khaira B. K., Wiegand I., Overhage J., Hancock R. E. (2008). Complex Ciprofloxacin Resistome Revealed by Screening a *Pseudomonas aeruginosa* Mutant Library for Altered Susceptibility. *Antimicrobial Agents and Chemotherapy*.

[11] Martinez J. L. (2009). Environmental Pollution by Antibiotics and by Antibiotic Resistance Determinants. *Environmental Pollution*.

[12] Jin D., Kong X., Cui B. (2018). Bacterial Communities and Potential Waterborne Pathogens Within the Typical Urban Surface Waters. *Scientific Reports*.

[13] World Health Organization (2021). *WHO Global Water, Sanitation and Hygiene: Annual Report 2020*.

[14] Patel C. B., Shanker R., Gupta V. K., Upadhyay R. S. (2016). Q-PCR Based Culture-Independent Enumeration and Detection of Enterobacter: An Emerging Environmental Human Pathogen in Riverine Systems and Potable Water. *Frontiers in Microbiology*.

[15] Yang Y., Zhang T., Zhang X. X. (2012). Quantification and Characterization of β-Lactam Resistance Genes in 15 Sewage Treatment Plants from East Asia and North America. *Applied Microbiology and Biotechnology*.

[16] Khan G. A., Berglund B., Khan K. M., Lindgren P. E., Fick J. (2013). Occurrence and Abundance of Antibiotics and Resistance Genes in Rivers, Canal and Near Drug Formulation Facilities–A Study in Pakistan. *PLoS One*.

[17] Berendonk T. U., Manaia C. M., Merlin C. (2015). Tackling Antibiotic Resistance: The Environmental Framework. *Nature Reviews Microbiology*.

[18] Endale H., Mathewos M., Abdeta D. (2023). Potential Causes of Spread of Antimicrobial Resistance and Preventive Measures in One Health Perspective-A Review. *Infection and Drug Resistance*.

[19] Pond M. J., Nori A. V., Witney A. A., Lopeman R. C., Butcher P. D., Sadiq S. T. (2014). High Prevalence of Antibiotic-Resistant Mycoplasma Genitalium in Nongonococcal Urethritis: The Need for Routine Testing and the Inadequacy of Current Treatment Options. *Clinical Infectious Diseases*.

[20] Lin L., Yuan K., Liang X. (2015). Occurrences and Distribution of Sulfonamide and Tetracycline Resistance Genes in the Yangtze River Estuary and Nearby Coastal Area. *Marine Pollution Bulletin*.

[21] Maestre‐Carballa L., Lluesma Gomez M., Angla Navarro A., Garcia‐Heredia I., Martinez‐Hernandez F., Martinez‐Garcia M. (2019). Insights Into the Antibiotic Resistance Dissemination in a Wastewater Effluent Microbiome: Bacteria, Viruses and Vesicles Matter. *Environmental Microbiology*.

[22] Mondal L., Hossain T., Saha M. L. (2024). Bacterial Load, Multiple Antibiotic-Resistance Patterns, and Cytotoxic Effects of Coliform and Coliform-Related Bacteria Associated With the Surface Water of Dhaka City. *Bangladesh Journal of Botany*.

[23] Haque M. F., Rani S. S., Kumar Saha A. (2019). Bacteriological Evaluation of Drinking Water of Rajshahi City, Bangladesh. *American Scientific Research Journal for Engineering, Technology, and Sciences (ASRJETS)*.

[24] Singh A. K., Das S., Kumar S. (2020). Distribution of Antibiotic-Resistant Enterobacteriaceae Pathogens in Potable Spring Water of Eastern Indian Himalayas: Emphasis on Virulence Gene and Antibiotic Resistance Genes in *Escherichia coli*. *Frontiers in Microbiology*.

[25] Fiaz M., Ahmed I., Riaz R., Nawaz U., Arshad M. (2021). Prevalence of Antibiotic-Resistant Bacterial Strains in Wastewater Streams: Molecular Characterization and Relative Abundance. *Folia Microbiologica*.

[26] Rashid A., Mirza S. A., Keating C., Ali S., Campos L. C. (2022). Indigenous *Bacillus paramycoides* spp. and *Alcaligenes faecalis*: Sustainable Solution for Bioremediation of Hospital Wastewater. *Environmental Technology*.

[27] Ibrahim I. A., Hameed T. A. (2015). Isolation, Characterization and Antimicrobial Resistance Patterns of Lactose-Fermenter Enterobacteriaceae Isolates From Clinical and Environmental Samples. *Open Journal of Medical Microbiology*.

[28] Delik E., Eroğlu B., Tefon-Öztürk B. E. (2024). Evaluation of the In Vitro Effects of Concentrations of Antibiotics on Three Enterobacteriaceae Isolates. *World Journal of Microbiology and Biotechnology*.

[29] Koskeroglu K., Barel M., Hizlisoy H., Yildirim Y. (2023). Biofilm Formation and Antibiotic Resistance Profiles of Water-Borne Pathogens. *Research in Microbiology*.

[30] Obayiuwana A., Ogunjobi A., Yang M., Ibekwe M. (2018). Characterization of Bacterial Communities and Their Antibiotic Resistance Profiles in Wastewaters Obtained From Pharmaceutical Facilities in Lagos and Ogun States, Nigeria. *International Journal of Environmental Research and Public Health*.

[31] Kim Y. J., Kim Y. G. (2016). Study on Antibiotic Resistant Bacteria in Surface Water Receiving Pharmaceutical Complex Effluent. *Korean Journal of Environmental Health Sciences*.

[32] Islam M. M., Iqbal M. S., Leemans R., Hofstra N. (2018). Modelling the Impact of Future Socio-Economic and Climate Change Scenarios on River Microbial Water Quality. *International Journal of Hygiene and Environmental Health*.

[33] Bergey D. H. (1994). *Bergey’s Manual of Determinative Bacteriology*.

[34] Mahmud S., Nazir K. H. M. N. H., Rahman M. (2018). Prevalence and Molecular Detection of Fluoroquinolone-Resistant Genes (qnrA and qnrS) in *Escherichia coli* Isolated From Healthy Broiler Chickens. *Veterinary World*.

[35] McCabe K. M., Zhang Y. H., Huang B. L., Wagar E. A., McCabe E. R. (1999). Bacterial Species Identification After DNA Amplification With a Universal Primer Pair. *Molecular Genetics and Metabolism*.

[36] Saitou N., Nei M. (1987). The Neighbor-Joining Method: A New Method for Reconstructing Phylogenetic Trees. *Molecular Biology and Evolution*.

[37] Tamura K., Nei M., Kumar S. (2004). Prospects for Inferring Very Large Phylogenies by Using the Neighbor-Joining Method. *Proceedings of the National Academy of Sciences*.

[38] Bauer A. W., Kirby W. M., Sherris J. C., Turck M. (1966). Antibiotic Susceptibility Testing by a Standardized Single Disk Method. *American Journal of Clinical Pathology*.

[39] CLSI (2016). *Performance Standards for Antimicrobial Susceptibility Testing: 17th Informational Supplement*.

[40] Sweeney M. T., Lubbers B. V., Schwarz S., Watts J. L. (2018). Applying Definitions for Multidrug Resistance, Extensive Drug Resistance and Pandrug Resistance to Clinically Significant Livestock and Companion Animal Bacterial Pathogens. *Journal of Antimicrobial Chemotherapy*.

[41] Krumperman P. H. (1983). Multiple Antibiotic Resistance Indexing of *Escherichia coli* to Identify High-Risk Sources of Fecal Contamination of Foods. *Applied and Environmental Microbiology*.

[42] Bartoš O., Chmel M., Swierczková I. (2024). The Overlooked Evolutionary Dynamics of 16S rRNA Revises Its Role as the “Gold Standard” for Bacterial Species Identification. *Scientific Reports*.

[43] Mudenda S., Chabalenge B., Daka V. (2023). Global Strategies to Combat Antimicrobial Resistance: A One Health Perspective. *Pharmacology & Pharmacy*.

[44] Marinescu F., Marutescu L., Savin I., Lazar V. (2015). Antibiotic Resistance Markers Among Gram-Negative Isolates from Wastewater and Receiving Rivers in South Romania. *Romanian Biotechnological Letters*.

[45] Stachurová T., Sýkorová N., Semerad J., Malachova K. (2022). Resistant Genes and Multidrug-Resistant Bacteria in Wastewater: A Study of Their Transfer to the Water Reservoir in the Czech Republic. *Life*.

[46] Cornacchia A., Centorotola G., Saletti M. A. (2021). Virulence and Antibiotic Resistance of *Klebsiella pneumoniae* Strains Isolated From Wastewater. *The European Journal of Public Health*.

[47] Liu M., Zheng L., Zhu L. (2023). Characteristics of Carbapenem-Resistant *Klebsiella pneumoniae* in Sewage From a Tertiary Hospital in Jilin Province, China. *PLoS One*.

[48] Lin J., Tang B., Zheng X. (2022). Emergence of Incl2 Plasmid-Mediated Colistin Resistance in Avian *Escherichia fergusonii*. *FEMS Microbiology Letters*.

