# Supplementary material for: Molecular Characterization and Antibiogram Profiling of Bacteria Isolated From Sewage and Surface Water in Bangladesh
Source: Scientifica (Cairo). 2025 Sep 3;2025:1848058. doi: 10.1155/sci5/1848058 (PMC12422855; doi:10.1155/sci5/1848058)
Supplement: Supporting Information — Additional supporting information can be found online in the Supporting Information section. [file 1848058.f1.docx]

***Supplementary Information***

***for***

**Molecular Characterization and Antibiogram Profiling of Bacteria Isolated from Sewage and Surface Water in Bangladesh**

Md. Arif-Uz-Zaman Polash^1^, Md. Shamsul Islam^1^, Nusrat Zahan^1^, Subir Sarker^2,3^, Md. Hakimul Haque^1,3^**^*^**


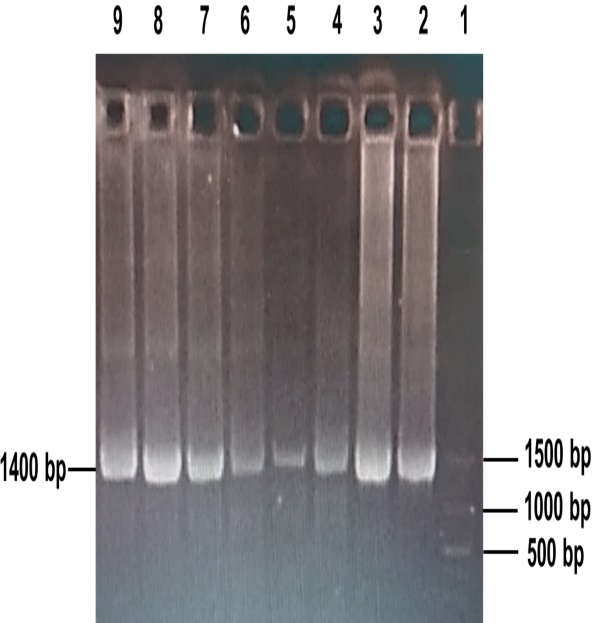


Figure S1. Amplification of the 16S rRNA gene in a 1.5% agarose gel. The 16S rRNA gene is present in all samples (Lanes 2–9). A 1 kb DNA ladder was used as a molecular weight marker for comparison.

**Table S1: Antimicrobial with zone of standard in diameter (according to the CLSI, 2016)**

| SL NO. | Antimicrobial agents | Symbol | Disc concentration.  (μg /disc) | Interpretation of results (zone of diameter in mm) | | |
| --- | --- | --- | --- | --- | --- | --- |
|  | | | | R | I | S |
| 1 | Amoxicillin + Clavulanic Acid | AMC | 30 | ≤13 | 14-17 | ≥18 |
| 2 | Amikacin | AK | 30 | ≤14 | 15-16 | ≥17 |
| 3 | Cefradine | CE | 30 | ≤12 | 13-17 | ≥18 |
| 4 | Ceftriaxone | CRO | 3O | ≤19 | 20-22 | ≥23 |
| 5 | Cefuroxime | CXM | 30 | ≤14 | 15-22 | ≥23 |
| 6 | Gentamycin | CN | 10 | ≤10 | 11-14 | ≥15 |
| 7 | Ciprofloxacin | CIP | 5 | ≤15 | 16-20 | ≥21 |
| 8 | Levofloxacin | LEV | 5 | ≤13 | 14-16 | ≥17 |
| 9 | Azithromycin | AZM | 15 | ≤13 | 14-17 | ≥18 |
| 10 | Imipenem | IPM | 10 | ≤21 | 22-24 | ≥25 |

**Table S2. Results of antimicrobial sensitivity and resistant pattern of *Klebsiella pneumoniae***

| **Antibiotic discs** | **Phenotypic isolates (n = 13)** | | |
| --- | --- | --- | --- |
|  | **Sensitive No. (%)** | **Intermediate No. (%)** | **Resistant No. (%)** |
| Amoxicillin + clavulanic acid | 2 (15.38%) | 2 (15.38%) | 9 (69.23%) |
| Amikacin | 8 (61.53%) | 2 (15.38%) | 3 (23.07%) |
| Cefradine | 2 (15.38%) | 1 (7.69%) | 10 (76.92%) |
| Ceftriaxone | 3 (23.07%) | 4 (30.77%) | 6 (46.15%) |
| Cefuroxime | 5 (38.46%) | 3 (23.07%) | 5 (38.46%) |
| Gentamicin | 5 (38.46%) | 2 (15.38%) | 6 (46.15%) |
| Ciprofloxacin | 4 (30.77%) | 1 (7.69%) | 8 (61.53%) |
| Levofloxacin | 9 (69.23%) | 2 (15.38%) | 2 (15.38%) |
| Azithromycin | 7 (53.84%) | 3 (23.07%) | 3 (23.07%) |
| Imipenem | 11 (84.61%) | 2 (15.38%) | 0(0%) |

**Table S3. Results of antimicrobial sensitivity and resistant pattern of *Klebsiella quasivariicola***

| **Antibiotic discs** | **Phenotypic isolates (n = 3)** | | |
| --- | --- | --- | --- |
|  | **Sensitive No. (%)** | **Intermediate No. (%)** | **Resistant No. (%)** |
| Amoxicillin + clavulanic acid | 1 (33.33%) | 0 (0%) | 2 (66.66%) |
| Amikacin | 2 (66.66%) | 1 (33.33%) | 0 (0%) |
| Cefradine | 0 (0%) | 1 (33.33%) | 2 (66.66%) |
| Ceftriaxone | 3 (100%) | 0 (0%) | 0 (0%) |
| Cefuroxime | 2 (66.66%) | 1 (33.33%) | 0 (0%) |
| Gentamicin | 2 (66.66%) | 0 (0%) | 1 (33.33%) |
| Ciprofloxacin | 2 (66.66%) | 1 (33.33%) | 0 (0%) |
| Levofloxacin | 3 (100%) | 0 (0%) | 0 (0%) |
| Azithromycin | 2 (66.66%) | 1 (33.33%) | 0 (0%) |
| Imipenem | 3 (100%) | 0 (0%) | 0 (0%) |

**Table S4. Results of antimicrobial sensitivity and resistant pattern of *Bacillus albus***

| **Antibiotic discs** | **Phenotypic isolates (n = 4)** | | |
| --- | --- | --- | --- |
|  | **Sensitive No. (%)** | **Intermediate No. (%)** | **Resistant No. (%)** |
| Amoxicillin + clavulanic acid | 0 (0%) | 2 (50%) | 2 (50%) |
| Amikacin | 3 (75%) | 1 (25%) | 0 (0%) |
| Cefradine | 0 (0%) | 1 (25%) | 3 (75%) |
| Ceftriaxone | 1 (25%) | 1 (25%) | 2 (50%) |
| Cefuroxime | 0 (0%) | 2 (50%) | 2 (50%) |
| Gentamicin | 2 (50%) | 1 (25%) | 1 (25%) |
| Ciprofloxacin | 2 (50%) | 1 (25%) | 1 (25%) |
| Levofloxacin | 3 (75%) | 1 (25%) | 0 (0%) |
| Azithromycin | 3 (75%) | 1 (25%) | 0 (0%) |
| Imipenem | 4 (100%) | 0 (0%) | 0 (0%) |

**Table S5. Results of antimicrobial sensitivity and resistant pattern of *Bacillus paramycoides***

| **Antibiotic discs** | **Phenotypic isolates (n = 5)** | | |
| --- | --- | --- | --- |
|  | **Sensitive No. (%)** | **Intermediate No. (%)** | **Resistant No. (%)** |
| Amoxicillin + clavulanic acid | 2 (40%) | 1 (20%) | 2 (40%) |
| Amikacin | 4 (80%) | 1 (20%) | 0 (0%) |
| Cefradine | 1 (20%) | 1 (20%) | 3 (60%) |
| Ceftriaxone | 2 (40%) | 1 (20%) | 2 (40%) |
| Cefuroxime | 3 (60%) | 1 (20%) | 1 (20%) |
| Gentamicin | 4 (80%) | 0 (0%) | 1 (20%) |
| Ciprofloxacin | 4 (80%) | 0 (0%) | 1 (20%) |
| Levofloxacin | 4 (80%) | 1 (20%) | 0 (0%) |
| Azithromycin | 4 (80%) | 1 (20%) | 0 (0%) |
| Imipenem | 5 (100%) | 0 (0%) | 0 (0%) |

**Table S6. Results of antimicrobial sensitivity and resistant pattern of *Lysinibacillus xylanilyticus***

| **Antibiotic discs** | **Phenotypic isolates (n = 3)** | | |
| --- | --- | --- | --- |
|  | **Sensitive No. (%)** | **Intermediate No. (%)** | **Resistant No. (%)** |
| Amoxicillin + clavulanic acid | 1 (33.33%) | 1 (33.33%) | 1 (33.33%) |
| Amikacin | 2 (66.66%) | 0 (0%) | 1 (33.33%) |
| Cefradine | 1 (33.33%) | 0 (0%) | 2 (66.66%) |
| Ceftriaxone | 2 (66.66%) | 0 (0%) | 1 (33.33%) |
| Cefuroxime | 3 (100%) | 0 (0%) | 0 (0%) |
| Gentamicin | 3 (100%) | 0 (0%) | 0 (0%) |
| Ciprofloxacin | 2 (66.66%) | 1 (33.33%) | 0 (0%) |
| Levofloxacin | 3 (100%) | 0 (0%) | 0 (0%) |
| Azithromycin | 2 (66.66%) | 1 (33.33%) | 0 (0%) |
| Imipenem | 3 (100%) | 0 (0%) | 0 (0%) |

**Table S7. Results of antimicrobial sensitivity and resistant pattern of *Enterobacter bugandensis***

| **Antibiotic discs** | **Phenotypic isolates (n = 8)** | | |
| --- | --- | --- | --- |
|  | **Sensitive No. (%)** | **Intermediate No. (%)** | **Resistant No. (%)** |
| Amoxicillin + clavulanic acid | 1 (12.5%) | 1 (12.5%) | 6 (75%) |
| Amikacin | 5 (62.5%) | 2 (25%) | 1 (12.5%) |
| Cefradine | 1 (12.5%) | 1 (12.5%) | 6 (75%) |
| Ceftriaxone | 3 (37.5%) | 0 (0%) | 5 (62.5%) |
| Cefuroxime | 1 (12.5%) | 2 (25%) | 5 (62.5%) |
| Gentamicin | 4 (50%) | 3 (37.5%) | 1 (12.5%) |
| Ciprofloxacin | 1 (12.5%) | 3 (37.5%) | 4 (50%) |
| Levofloxacin | 7 (87.5%) | 1 (12.5%) | 0 (0%) |
| Azithromycin | 3 (37.5%) | 2 (25%) | 3 (37.5%) |
| Imipenem | 8 (100%) | 0 (0%) | (0%) |

**Table S8. Results of antimicrobial sensitivity and resistant pattern of *Escherichia fergusonii***

| **Antibiotic discs** | **Phenotypic isolates (n = 9)** | | |
| --- | --- | --- | --- |
|  | **Sensitive No. (%)** | **Intermediate No. (%)** | **Resistant No. (%)** |
| Amoxicillin + clavulanic acid | 1 (11.11%) | 2 (22.22%) | 6 (66.66%) |
| Amikacin | 5 (55.55%) | 2 (22.22%) | 2 (22.22%) |
| Cefradine | 1 (11.111%) | 1 (11.11%) | 7 (77.77%) |
| Ceftriaxone | 2 (22.22%) | 3 (33.33%) | 4 (44.44%) |
| Cefuroxime | 2 (22.22%) | 3 (33.33%) | 4 (44.44%) |
| Gentamicin | 4 (44.44%) | 3 (33.33%) | 2 (22.22%) |
| Ciprofloxacin | 3 (33.33%) | 3 (33.33%) | 3 (33.33%) |
| Levofloxacin | 7 (77.77%) | 1 (11.11%) | 1 (11.11%) |
| Azithromycin | 4 (44.44%) | 1 (11.11%) | 4 (44.44%) |
| Imipenem | 9 (100%) | 0 (0%) | 0 (0%) |

**Table S9. Results of antimicrobial sensitivity and resistant pattern of *Comamonas jiangduensis***

| **Antibiotic discs** | **Phenotypic isolates (n = 5)** | | |
| --- | --- | --- | --- |
|  | **Sensitive No. (%)** | **Intermediate No. (%)** | **Resistant No. (%)** |
| Amoxicillin + clavulanic acid | 1 (20%) | 2 (40%) | 2 (40%) |
| Amikacin | 5 (100%) | 0 (0%) | 0 (0%) |
| Cefradine | 1 (20%) | 1 (20%) | 3 (60%) |
| Ceftriaxone | 4 (80%) | 1 (20%) | 0 (0%) |
| Cefuroxime | 3 (60%) | 2 (40%) | 0 (0%) |
| Gentamicin | 4 (80%) | 1 (20%) | 0 (0%) |
| Ciprofloxacin | 3 (60%) | 1 (20%) | 1 (20%) |
| Levofloxacin | 5 (100%) | 0 (0%) | 0 (0%) |
| Azithromycin | 4 (80%) | 1 (20%) | 0 (0%) |
| Imipenem | 5 (100%) | 0 (0%) | 0 (0%) |
